# Different sources of alfalfa hay alter the composition of rumen microbiota in mid-lactation Holstein cows without affecting production performance

**DOI:** 10.3389/fvets.2024.1433876

**Published:** 2024-10-21

**Authors:** Shaokai La, Hao Li, Yan Zhang, Muhammad Abaidullah, Jiakuan Niu, Zimin Gao, Boshuai Liu, Sen Ma, Yalei Cui, Defeng Li, Yinghua Shi

**Affiliations:** ^1^College of Animal Science and Technology, Henan Agricultural University, Zhengzhou, China; ^2^Henan Key Laboratory of Innovation and Utilization of Grassland Resources, Zhengzhou, China; ^3^Henan Forage Engineering Technology Research Center, Zhengzhou, China

**Keywords:** alfalfa hay, lactation performance, ruminal fermentation, microbiota, dairy cow

## Abstract

Alfalfa hay is a commonly used and important feed ingredient in dairy production. To better expand the alfalfa supply market, it is of great significance to explore the impact of alfalfa hay from different sources on dairy cow production performance. This study compared the effects of imported alfalfa hay from America (AAH) and Spain (SAH) on lactation performance and rumen microbiota of cows. Three hundred and sixty healthy mid-lactation Holstein cows with similar body weight, milk yield, and parity were randomly divided into two groups fed diets based on AAH or SAH for a 70-day experimental period. Each group was composed of four pens, with 45 cows in each pen. Daily records were kept for MY per cow and dry matter intake per pen. Twelve randomly selected cows per group were sampled to collect milk, feces, rumen fluid, and blood. The findings revealed no significant differences between the two groups in terms of production performance, nutrient apparent digestibility, serum biochemical indices, or rumen fermentation parameters. However, rumen microbial composition differed significantly between the two groups of cows based on β-diversity. On the genus level, the relative abundance of *Prevotella*, *Succinivibrionaceae_UCG-002* increased while that of *NK4A214_group*, *Ruminococcus*, *norank_f_F082* and *Lachnospiraceae_NK3A20_group* decreased in the SAH group compared with AAH group. There was no significant correlation between these core differential bacteria and the molar proportions of acetate and propionate, the concentration of total volatile fatty acids, and milk yield. In conclusion, the feeding effects of SAH were similar to those of AAH. These findings provided a reference for the application of alfalfa hay from different sources and for the improvement of the economic benefit of dairy farms.

## 1 Introduction

Alfalfa is widely used in animal husbandry due to its good palatability, low fiber content and high protein concentration (ranging from 17 to 22%) (1). Feeding alfalfa can effectively reduce the proportion of concentrates, especially that of high-protein ingredients in diets (2). Therefore, alfalfa has become an irreplaceable forage in dairy farming, and the beneficial impact of alfalfa hay on the milk performance of dairy cows has been widely recognized.

The demand for premium alfalfa hay has increased significantly in recent years due to a rise in the number of dairy cows and an improvement in the milk yield (MY) of dairy cows (3). Typically, dairy farms rely on multiple suppliers for their alfalfa, which contributes to the stable operation of the farms. Dairy farmers need to select alfalfa hay from different sources based on factors such as price to maximize their benefits. Especially since the outbreak of the COVID-19 pandemic, the price and supply stability of alfalfa hay were challenged due to transportation issues and other factors. As a result, the substitution of alfalfa hay from different sources had become more frequent. It is well known that the impacts of alfalfa on production performance in dairy cows are not fixed, as its nutrient composition is variable due to breed (4), storage methods (5) and harvest period. For example, Vagnoni and Broderick (6) found that more crude protein was degraded in the rumen in cows given alfalfa silage than in those given alfalfa hay, while cows given alfalfa hay had greater dry matter intake (DMI). Additionally, previous studies have conducted trials comparing alfalfa hay with other forages such as wheat straw and peanut seedlings (7–9). However, there have been few studies that compare how alfalfa hay from various sources affects dairy cows. Despite similar nutritional profiles, the frequent changes of alfalfa hay from various sources, especially those from different countries, have raised concerns among dairy farmers due to the potential threat to the stability of dairy cow production performance.

Ruminants possess a unique physiological structure that synergistically interacts with rumen bacteria to facilitate the fermentation and degradation of forage into volatile fatty acids (VFA). These VFA serve as a vital source of nutrition for ruminants (10). Indeed, the fermentation of fiber in the rumen produces two different lipogenic VFA: acetate and butyrate (11). A significant portion of butyrate is converted into β-hydroxybutyrate within the rumen wall tissue. This acetate and β-hydroxybutyrate serve as a substrate for about half of the fat found in milk. Alfalfa hay, as a high-protein forage, plays a crucial role in rumen microorganisms and thus affects dairy cow production performance (12).

Therefore, this experiment compared the effects of different sources of alfalfa hay imported from America (AAH) or Spain (SAH) with similar nutrient composition on lactation performance, nutrient apparent digestibility, serum biochemical indices, ruminal fermentation, and microbiota of dairy cows. We hypothesize that alfalfa hay from different sources with similar nutritional profiles will not impact dairy cow production performance, providing dairy farmers with more options to enhance the economic efficiency of the dairy farm.

## 2 Materials and methods

### 2.1 Alfalfa hay

The alfalfa hay used in this study, obtained from Spain and the US, respectively, was bought from Literana, LLC and Stone Wings II, LLC. We purchased 60 tons each of American and Spanish alfalfa hay, with 60 bales of each. We randomly selected 10 bales, and collected 500 g samples from the upper, middle and lower layers of each bundle, and mixed them for the determination of nutritional composition. Table 1 shows the nutrients present in alfalfa hay.

**Table 1:** Nutrient composition of alfalfa hay.

Item	AAH^a^	SAH^a^
Dry matter (DM)	90.52	91.92
Crude protein (% DM)	16.39	17.74
Neutral detergent fiber (% DM)	32.17	35.52
Acid detergent fiber (% DM)	24.35	26.53
Ether extract (% DM)	1.72	1.63
Calcium (% DM)	2.03	1.65
Phosphorus (% DM)	0.29	0.30
Relative feed value^b^	202.2	178.7

a AAH, American alfalfa hay; SAH, Spanish alfalfa hay.

b Relative feed value was calculated based on the contents of acid detergent fiber and neutral detergent fiber.

### 2.2 Cows, experimental design and diets

This study was approved by the Institutional Animal Care and Use Committee of Henan Agriculture University (Zhengzhou, China) (Approval Number: HNND2021062812). The trial was conducted in Xincai Ruiya Animal Husbandry Farm, Henan Province. Three hundred and sixty healthy mid-lactation Holstein dairy cows (MY = 21.98 ± 5.02 kg; days in milk = 208 ± 19.42 d; parity = 2.34 ± 0.47, mean ± SD) were randomly assigned to two groups (four replicates in each group and 45 cows in each replicate) fed diets based on AAH or SAH. The trial lasted 70 days, with 10 days for adaptation and 60 days for collecting data and samples. Dietary composition and nutrient levels are shown in Table 2. All cows were fed twice daily (07:00 and 19:00 h) and milked 3 times (06:30, 14:30, and 22:30 h) a day, and given access to water at all times. The barn was cleaned and disinfected once a week.

**Table 2:** Ingredient and chemical composition of experimental diet.

Item	AAH^a^	SAH^a^
**Ingredients (% DM)**		
Corn silage	31.11	31.09
Brewer’s grain	4.00	4.00
Oat hay	5.00	5.00
Corn grain, ground	7.30	7.29
Alfalfa hay	12.83	12.88
Soybean hull	4.45	4.45
Soybean meal	4.24	4.24
Cottonseed cake	4.34	4.33
Sodium bicarbonate	0.71	0.71
Concentrate supplement^b^	26.02	26.01
**Chemical composition (% DM)**		
Crude protein	14.91	14.93
Neutral detergent fiber	47.10	47.00
Acid detergent fiber	19.31	20.34
Calcium	0.82	0.89
Phosphorus	0.38	0.38
NEL^c^ (MJ/kg)	6.66	6.65

a AAH, American alfalfa hay; SAH, Spanish alfalfa hay.

b Contained per kg premix dry matter: 31.3 mg Co, 343.5 mg Cu, 2, 258 mg Fe, 1, 160 mg Mn, 1, 534 mg Zn, 40.3 mg I, 17.7 mg Se, 317.4 KIU vitamin A, 80.8 KIU vitamin D, and 3, 030 IU vitamin E.

c NEL, net energy for lactating cow.

### 2.3 Collection of data and samples

During the collection period, the feed offered and rejected for each replication was noted daily to determine DMI. The MY of each of the 360 experimental cows was recorded daily. In addition, total mixed ration (TMR) samples were collected daily. After the experiment ended, the TMR samples were mixed in proportion for determining the nutritional composition of TMR. To determine whether there were differences in milk composition, apparent nutrient digestibility, and rumen microbiota between the two groups of cows, 12 cows were randomly selected from each group (three cows were in each repetition) for samples of milk, feces, blood, and rumen fluid. Milk samples were collected on day 0, 14, 28, 42, 56, and the last 4 days of the experiment. For each cow, 40 mL of milk samples was collected per collection day (in a 4:3:3 ratio in the morning, midday, and evening) and placed in a centrifuge tube containing potassium dichromate preservative. The milk samples were refrigerated at 4°C for milk composition analysis. Fecal samples were collected 4 times a day (06:00, 12:00, 18:00, and 24:00 h) during the last 4 days of the experiment. Approximately 250 g of feces were collected per cow each time using a rectal sampling method, mixed, and 120 g of the fecal samples were weighed and mixed with 30 mL of 10% tartaric acid. The feces samples collected over the 4 d were separately mixed at the end of the experiment for chemical analysis. Blood was collected through the caudal vein with 5 mL vacuum tubes before morning feeding on the last day of the trial. The blood was immediately sent to the laboratory and centrifuged at 3,000 × g for 15 min at 4°C. And then serum was gathered into 2 mL centrifuge tubes and kept at −20°C until assayed. Rumen fluid was collected on the last day of the trial using a gastric tube with an exterior diameter of 1 cm, an internal diameter of 0.8 cm, and a length of 300 cm, 2 h after morning feeding. To prevent saliva pollution, about 200 mL of rumen fluid from individual cows was removed and discarded. Next, 200 mL of rumen fluid was collected in a 500 mL beaker and immediately sent to the laboratory for pH analysis using a PHS-10 meter (Sartorius, Göttingen, Germany) and recording. After filtration through 4 layers of gauze, the rumen fluid was divided into 3 portions, each containing 10 mL. These samples were then frozen and stored at −80°C for subsequent determination of VFA and ammonia (NH_3_-N), as well as for 16S rRNA sequencing.

### 2.4 Analytical methods

The concentrations of dry matter (DM), ether extract and acid detergent fiber in alfalfa hay, feces and TMR samples, as well as the acid insoluble ash in feces and TMR samples, were determined using the methods described in AOAC (13). The concentration of neutral detergent fiber in alfalfa hay, feces, and TMR samples was determined using the method of Van Soest et al. (14). The concentrations of crude protein and nitrogen were determined using an automatic Kjeldahl N analyzer (SKD-2000, Haineng Experimental Instrument Technology Co., Ltd., Shanghai, China). The concentrations of calcium and phosphorus in alfalfa hay and TMR were analyzed based on the method of Mattioli et al. (15). Acid insoluble ash was used as an endogenous indicator, according to the formula: the apparent digestibility of a nutrient (%) = 100 − 100 × the nutrient concentration in feces (%) × acid insoluble ash concentration in TMR (%)/the nutrient concentration in TMR (%)/acid insoluble ash concentration in feces (%), calculating the apparent digestibility of crude protein, ether extract, acid detergent fiber, and neutral detergent fiber.

An automated near-infrared milk analyzer (Seris300 CombiFOSS; Foss Electric, Hillerd, Denmark) was employed to measure the concentrations of milk fat, protein, lactose, milk solids, milk urea nitrogen (MUN), and somatic cell count in milk samples delivered to the Dairy Herd Improvement Testing Center in Henan Province. Serum concentrations of glucose, total protein, albumin, globulin, triglyceride, cholesterol, non-esterified fatty acids, β-hydroxybutyrate, urea, alanine aminotransferase and aspartate aminotransferase were assessed using commercially available assay kits obtained from Nanjing Jian Cheng Biological Technology Co. (Nanjing, Jiangsu, China).

For the rumen fluid samples used for the determination of VFA, after thawing at 4°C, they were treated with 25% metaphosphoric acid. Then, the concentrations of VFA (acetate, propionate, butyrate, isobutyrate, valerate, and isovalerate) were determined using a German Sykam ion chromatograph and a Dionex AS11-HC column (4 × 250 mm, Thermo Fisher Science, Waltham, MA, United States). The following chromatographic circumstances applied: a column temperature of 30°C, a flow rate of 1 mL/min, and an injection volume of 10 μL; the mobile phase was 0.1 mmol/L and 50 mmol/L NaOH solutions. The former was maintained for 28 min and the latter for 5 min. Finally, 0.1 mmol/L of NaOH solution was maintained for 10 min. For the rumen fluid samples used for the determination of NH_3_-N, after thawing at 4°C, the NH_3_-N concentration was determined using a UV-2100 spectrophotometer (UV2100, Shanghai Younike Instrument Co., Ltd., Shanghai, China) according to AOAC (13).

### 2.5 DNA extraction and 16S rRNA gene sequencing

Six rumen fluid samples were randomly selected from each group for DNA extraction and 16S rRNA gene sequencing. According to the manufacturer’s instructions, total bacterial DNA from rumen fluid was obtained using the E.Z.N.A.^®^ soil DNA Kit (Qiagen, United States). The NanoDrop-2000 UV–vis spectrophotometer (Thermo Scientific, Wilmington, United States) was used to detect DNA concentrations. Additionally, 1% agarose gel electrophoresis was used to evaluate DNA quality. The bacterial V3–V4 region of the 16S rRNA gene was amplified using primers for 338F (5′-ACTCCTACGGGAGGCAGCAG-3′) and 806R (5′-GGACTACHVGGGTWTCTAAT-3′) at a temperature of 55°C, as previously described (16). Following purification and quantification, PCR products were used by Majorbio Bio-Pharm Technology Co., Ltd. (Shanghai, China) to build libraries and sequence them on the Illumina MiSeq PE300 platform/NovaSeq PE250 platform (Illumina, San Diego, United States) in accordance with the established protocols. All results were based on sequenced reads and operational taxonomic units. The BioProject accession number for the 16S rRNA sequencing data, which was uploaded to the NCBI database, is PRJNA898964.

### 2.6 Processing of sequencing data

The optimized sequences were obtained by double-end sequence quality control splicing of sequencing results (17) and using FLASH version 1.2.7 (18). The optimised sequences were based on the silva138/16s_bacteria species classification database and subjected to operational taxonomic unit (OTU) clustering analysis (confidence level 0.7) at 97% similarity using UPARSE version 7.1 (19, 20), and a table of OTU species classification statistics was generated by drawing parity at the minimum number of sample sequences. Species number analysis, Alpha diversity analysis (Chao1 and Simpson indices), Beta diversity analysis (principal co-ordinates analysis), community composition analysis and mapping were carried out on the online tool of Majorbio Cloud Platform.^1^ Venn diagrams, which visually display the numbers of common and unique OTUs among groups, and the rarefaction curve (Shannon index) were drawn by R software (version 3.3.1). For the purpose of illustrating the variety and abundance of microbial communities, Chao1 and Simpson indices were calculated by Mothur (version 1.30.2) (21). Subsequently, the Bray-Curtis distance was calculated using Qiime software (version 1.9.1), and the principal co-ordinates analysis plot was generated using R software (version 3.3.1). The Vegan package based on the R software (Version 3.6.0) was used to test the differences in the microbial community structure among different treatments through ANOSIM. The relative abundance of phyla and genera was shown by barplot and heatmap, which were drawn by R software (version 3.3.1). The Wilcoxon rank-sum test method was used to analyze and identify the differences between the two groups. To calculate the impact of species abundance on the difference effect at the genus level, linear discriminant analysis effect size (LEfSe) and linear discriminant analysis were used. Spearman correlation coefficients were calculated between the top 20 species at the genus level and various ruminal fermentation parameters, as well as milk yield and milk composition using R language vegan package (version 3.3.1) and displayed on the heatmap. To predict functions of the ruminal microbiota, PICRUSt2 predictions of function were obtained based on the KEGG database.^2^ The differences in KEGG pathways between two groups were assessed by two-sided Welch’s t-test using STAMP software (version 2.1.3). Storey’s false discovery rate method was used for multiple test corrections as recommended by the STAMP developers. The top 20 important biological information (functional) was selected by sorting based on effect sizes (22).

### 2.7 Statistical analysis

Daily MY of 360 cows and DMI per replicate throughout the experiment were used for statistical analysis. Data from 24 cows used for sample collection showed no outliers (data points outside ±3 standard deviations from the mean), and their MY, milk composition, blood biochemical parameters, and rumen fermentation parameters were used for statistical analysis. The measurements of milk composition, blood biochemical parameters, and rumen fermentation parameters were repeated at least 3 times. The normality test and the variance homogeneity test were carried out using the PROC UNIVARIATE model and the PROC DISCRIM model, respectively. According to the characteristics of the data, one - way ANOVA or nonparametric test was performed using the PROC MIXED model, and the results were expressed as means. *p*-values >0.05 and <0.10 were considered a significant trend, while *p*-values <0.05 were considered significant.

## 3 Results

### 3.1 Influence on production performance and nutrient digestibility

**Figure 1:**
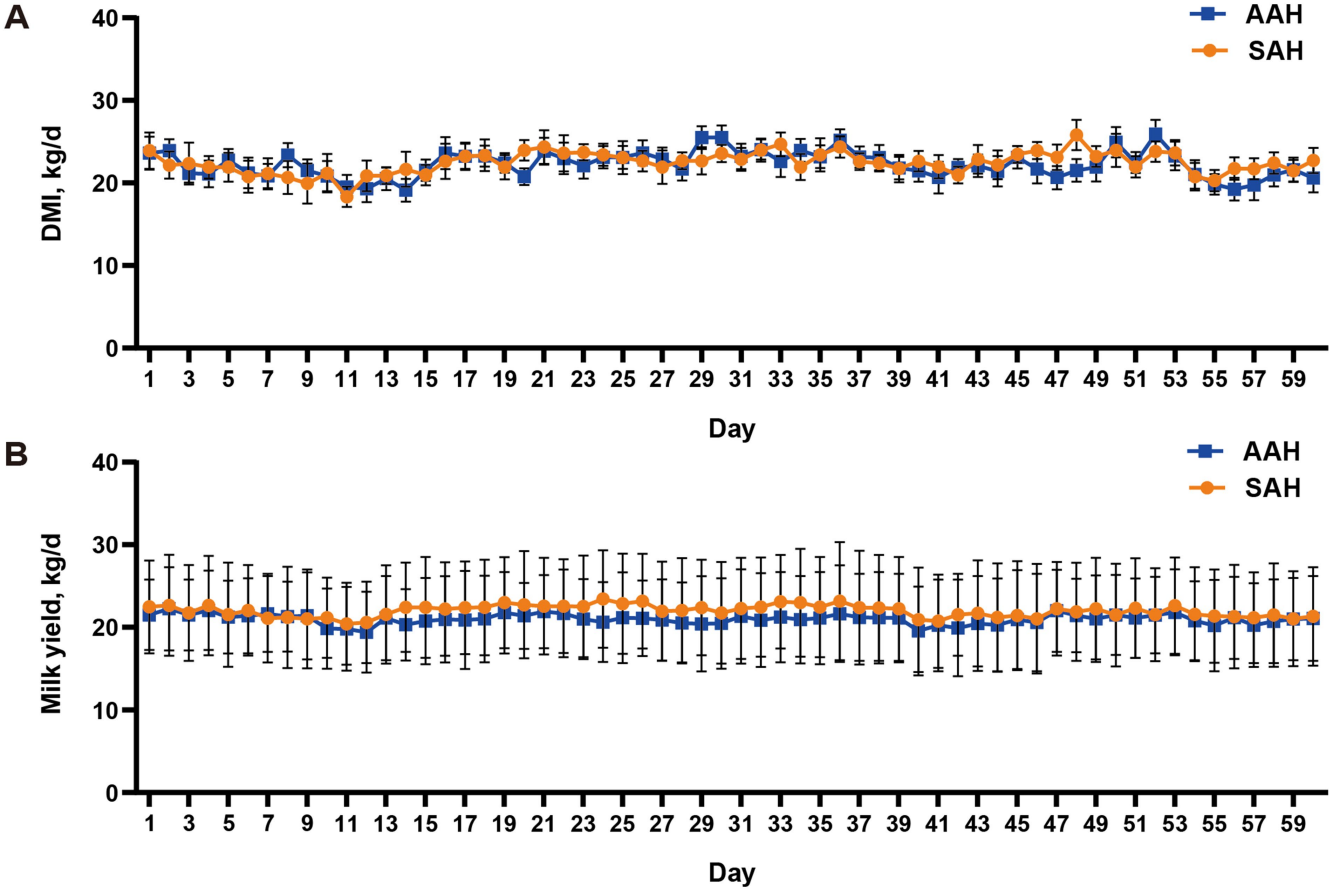
Comparison of different sources of alfalfa hay on dry matter intake [DMI, **(A)**] and milk yield [MY, **(B)**] in dairy cows. AAH, American alfalfa hay; SAH, Spanish alfalfa hay.

**Figure 2:**
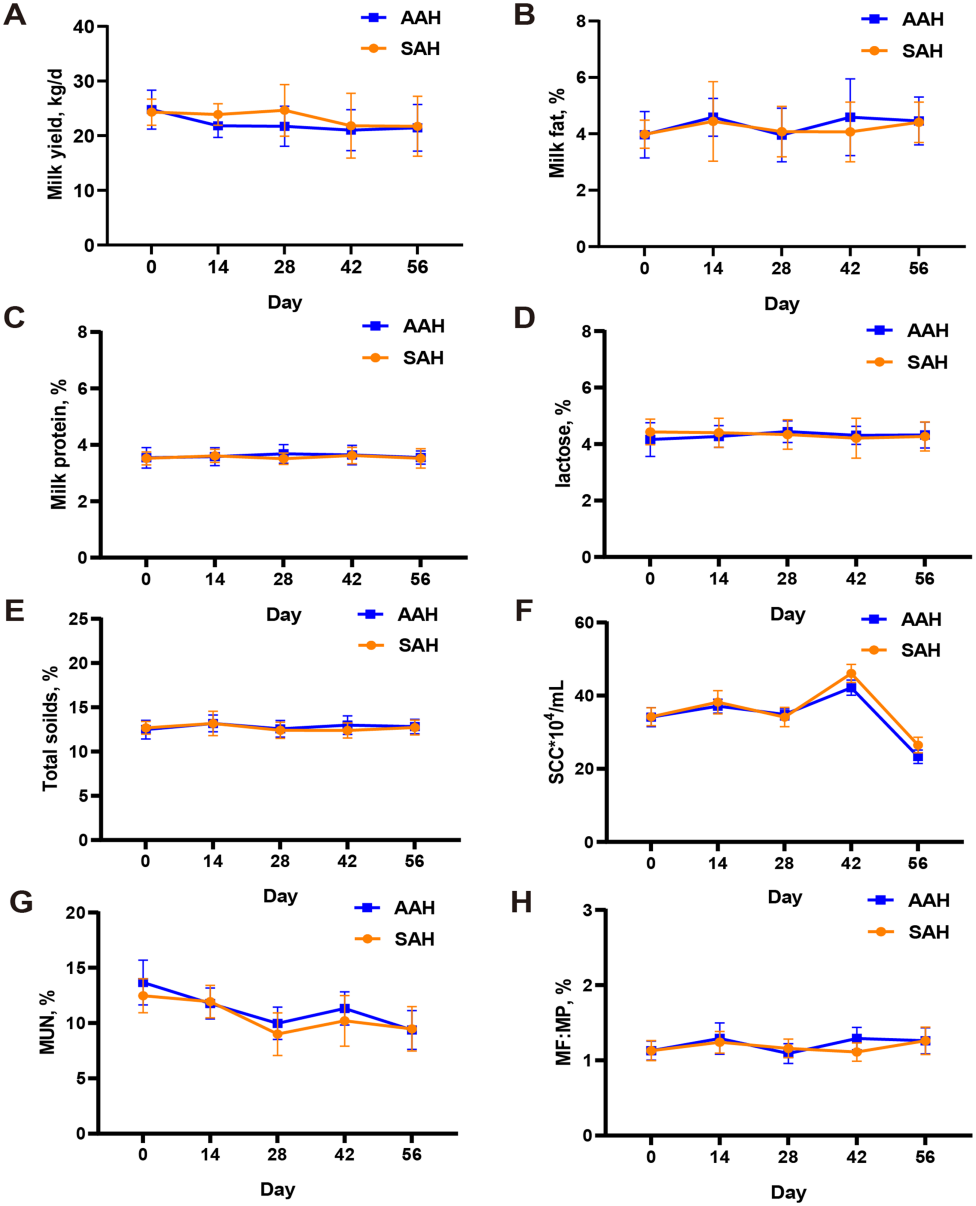
Comparison of different sources of alfalfa hay on milk yield and milk composition in dairy cows. **(A)** Milk yield. **(B)** Milk fat. **(C)** Milk protein. **(D)** Lactose. **(E)** Total soilds. **(F)** Somatic cell count (SCC). **(G)** Milk urea nitrogen (MUN). **(H)** The ratio of milk fat to milk protein (MF:MP). AAH, American alfalfa hay; SAH, Spanish alfalfa hay. Milk composition analysis in milk simples of AAH and SAH cows on days 0, 14, 28, 42 and 56 of the trial.

During the experiment, it was observed that alfalfa hay from different sources had no significant effect on the DMI (Figure 1A) and daily MY (Figure 1B) of the two groups of dairy cows (*p* > 0.05). This consistency was maintained throughout the entire collection period. Similarly, in the final 4 days of the experiment, there were no differences in MY among the cows used for sample collection (*p* > 0.05, Table 3). Furthermore, the analysis revealed no differences in milk components between the two dairy cow groups on specific days of the collection period, including days 14, 28, 42, 56, and the last 4 days (*p* > 0.05, Figure 2 and Table 3). This finding also extended to the apparent digestibility of nutrients, indicating that the source of alfalfa hay had no significant impact on this aspect as well (*p* > 0.05, Table 3).

**Table 3:** Comparison of different sources alfalfa hay on lactation performance and nutrient digestibility in dairy cows.

Item	Treatments^a^		SEM^b^	*p*-value
	AAH	SAH		
Milk yield (kg/d)	21.02	21.26	0.916	0.899
**Milk composition (%)**				
Milk protein (MP)	3.77	3.67	0.761	0.537
Milk fat (MF)	3.84	3.78	0.154	0.839
Lactose	4.70	4.50	0.126	0.428
Total solids	13.05	12.66	0.192	0.327
MUN^c^ (mg/dL)	13.40	13.68	0.410	0.745
SCC^d^ (×10^4^/mL)	22.84	25.10	3.986	0.785
MF:MP^e^	1.03	1.03	0.044	0.959
**Nutrient digestibility (%)**				
Dry matter	70.24	68.28	1.899	0.270
Organic matter	72.78	70.75	1.959	0.302
Crude protein	76.25	74.20	1.420	0.421
Ether extract	80.49	78.97	2.291	0.757
Neutral detergent fiber	71.56	69.58	1.469	0.190
Acid detergent fiber	55.33	48.70	2.258	0.150

a AAH, American alfalfa hay; SAH, Spanish alfalfa hay.

b SEM, standard error of mean.

c MUN, milk urea nitrogen.

d SCC, somatic cell count.

e MF:MP, the ratio of milk fat to milk protein.

### 3.2 Influence on blood biochemical indicators and rumen fermentation parameters

The serum concentrations of glucose, total protein, albumin, globulin, triglycerides, cholesterol, non-esterified fatty acids, β-hydroxybutyrate, and urea, as well as the activities of alanine aminotransferase and aspartate aminotransferase, showed no significant differences between the two groups of dairy cows (*p* > 0.05, Table 4). Similarly, rumen pH remained within the normal range and did not vary significantly between the groups (*p* > 0.05, Table 5). The rumen fermentation parameters were generally similar between the two groups, with the exception of isovalerate molar proportion, which was significantly higher in the SAH group compared to the AAH group (*p* = 0.007). Other fermentation parameters did not show statistically significant differences (*p* > 0.05).

**Table 4:** Comparison of different sources alfalfa hay on serum biochemical indices in dairy cows.

Item	Treatments^a^		SEM^b^	*p*-value
	AAH	SAH		
Glucose (mmol/L)	3.17	3.25	0.103	0.726
Total protein (g/L)	77.4	82.0	1.738	0.227
Albumin (g/L)	40.0	36.1	0.979	0.078
Globulin (g/L)	37.4	45.8	2.587	0.139
Albumin/Globulin	1.08	0.82	0.064	0.068
Triglyceride (mmol/L)	0.198	0.160	0.011	0.124
Cholesterol (mmol/L)	4.96	4.50	0.314	0.477
Non-esterified fatty acid (mmol/L)	0.110	0.103	0.010	0.755
β-hydroxybutyrate (mmol/L)	0.708	0.633	0.033	0.291
Urea (mmol/L)	5.40	5.07	0.217	0.474
Alanine transaminases (U/L)	28.4	27.7	1.714	0.835
Aspartate transaminase (U/L)	77.6	97	7.577	0.233
AST/ALT^c^	2.82	3.33	0.184	0.198

a AAH, American alfalfa hay; SAH, Spanish alfalfa hay.

b SEM, standard error of mean.

c AST/ALT, alanine transaminase/aspartate transaminase.

**Table 5:** Comparison of different sources alfalfa hay on ruminal fermentation in dairy cows.

Item	Treatments^1^		SEM^2^	*p*-value
	AAH	SAH		
pH	6.51	6.56	0.068	0.731
Total VFA^3^ (mM)	49.68	55.49	2.113	0.199
**Mol/100 mol**				
Acetate (A)	62.01	60.97	0.689	0.346
Propionate (P)	21.76	22.29	0.457	0.574
Butyrate	13.65	13.79	0.331	0.265
Valerate	1.63	1.71	0.084	0.194
Isobutyrate	0.291^b^	0.526^a^	0.034	0.007
Isovalerate	0.660	0.703	0.054	0.698
A:P^4^	2.88	2.74	0.090	0.467
Ammonia N (mg/100 mL)	9.33	11.27	1.217	0.460

^a,b^Means with different superscripts in each row differ significantly (*p* < 0.05).

1 AAH, American alfalfa hay; SAH, Spanish alfalfa hay.

2 SEM, standard error of mean.

3 VFA, volatile fatty acids.

4 A:P, acetate/propionate.

### 3.3 Influence on the phylum-level diversity and composition of the rumen microbiota

**Figure 3:**
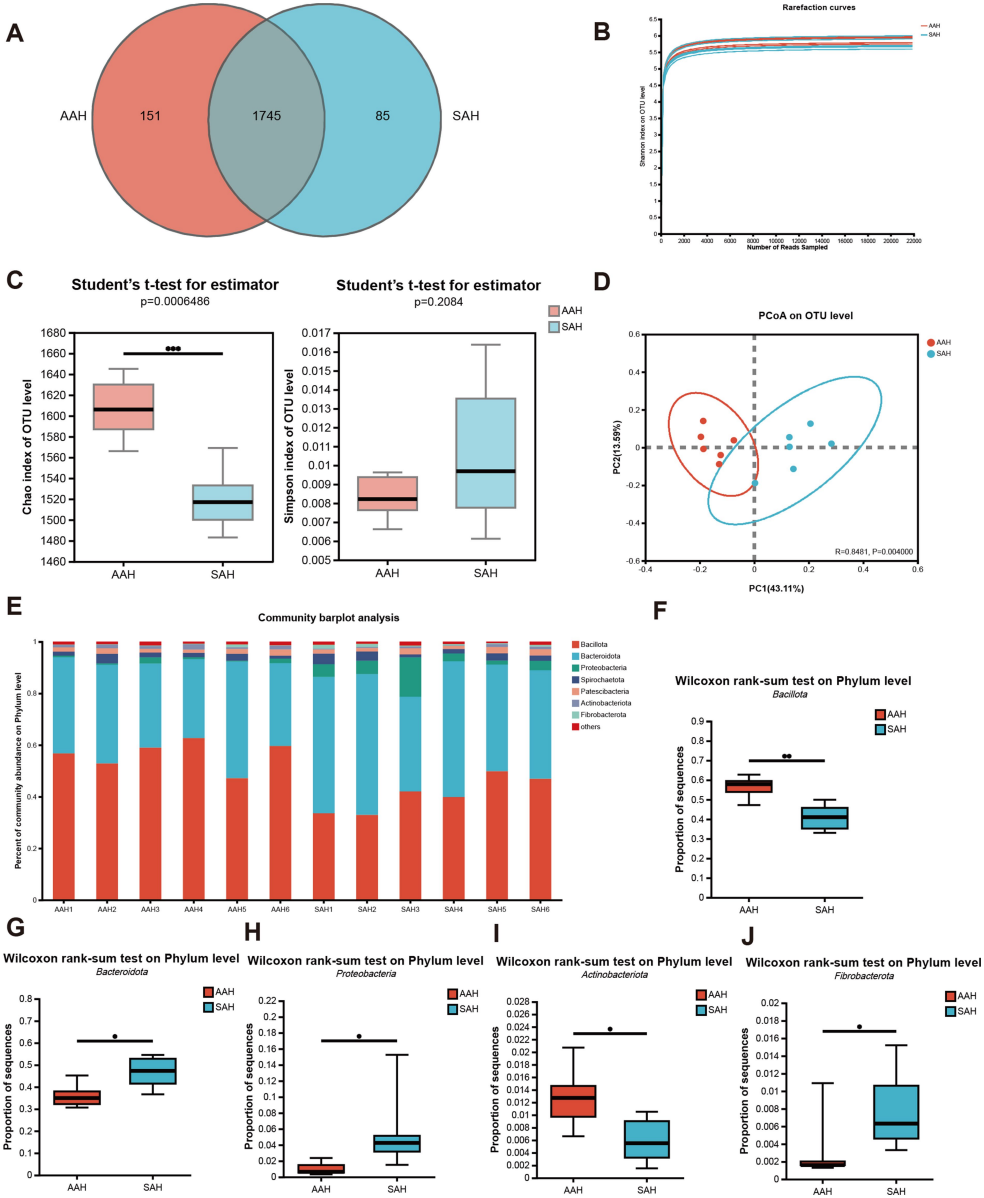
Comparison of different sources of alfalfa hay on ruminal microbiota in dairy cows. **(A)** Venn diagram of rumen microbiota on OTU level in the two groups. **(B)** Curve of rarefaction for sequencing data. **(C)** Alpha diversity. **(D)** Beta diversity. **(E)** Changes and differences in microbiome on phylum level. **(F–J)** Differential expression of ruminal microbes: **(F)** *Bacillota*; **(G)** *Bacteroidota*; **(H)** *Proteobacteria*; **(I)** *Actinobacteriota*; **(J)** *Fibrobacterota*. AAH, American alfalfa hay; SAH, Spanish alfalfa hay. * 0.01 < *p* ≤ 0.05; ** 0.001 < *p* ≤ 0.01.

The analysis of 12 rumen fluid samples resulted in 602,999 high-quality sequences after quality control, with an average sequence length of 417 bp and 251,558,244 bases. Taxonomic analysis at a 97% similarity threshold identified 1,981 OTUs across various taxonomic levels: Domain (1), Kingdom (1), Phylum (17), Class (34), Order (86), Family (149), Genus (304), and Species (589). Among these, the two groups together had 1,745 OTUs, with 151 unique to the AAH group and 85 unique to the SAH group (Figure 3A). The alpha diversity, assessed using the Shannon index, approached a plateau, indicating sufficient sequencing depth (Figure 3B). The Chao1 index was significantly higher in the AAH group compared to the SAH group (*p* < 0.05, Figure 3C), while the Simpson index showed no significant difference between the groups (*p* > 0.05, Figure 3C). The principal co-ordinates analysis plot revealed distinct clustering of the AAH and SAH groups (Figure 3D). In terms of phylum composition, both groups were dominated by *Bacillota* and *Bacteroidota*, which together constituted over 86% of the microbiota in each group (Figure 3E). Significant differences were observed between the groups: the SAH group had higher relative abundances of *Bacteroidota* (Figure 3G), *Proteobacteria* (Figure 3H), and *Fibrobacterota* (Figure 3J) (*p* < 0.05), but lower relative abundances of *Bacillota* (Figure 3F) and *Actinobacteria* (Figure 3I) (*p* > 0.05) compared to the AAH group.

### 3.4 Influence on composition and difference at the genus level of rumen microbiota

**Figure 4:**
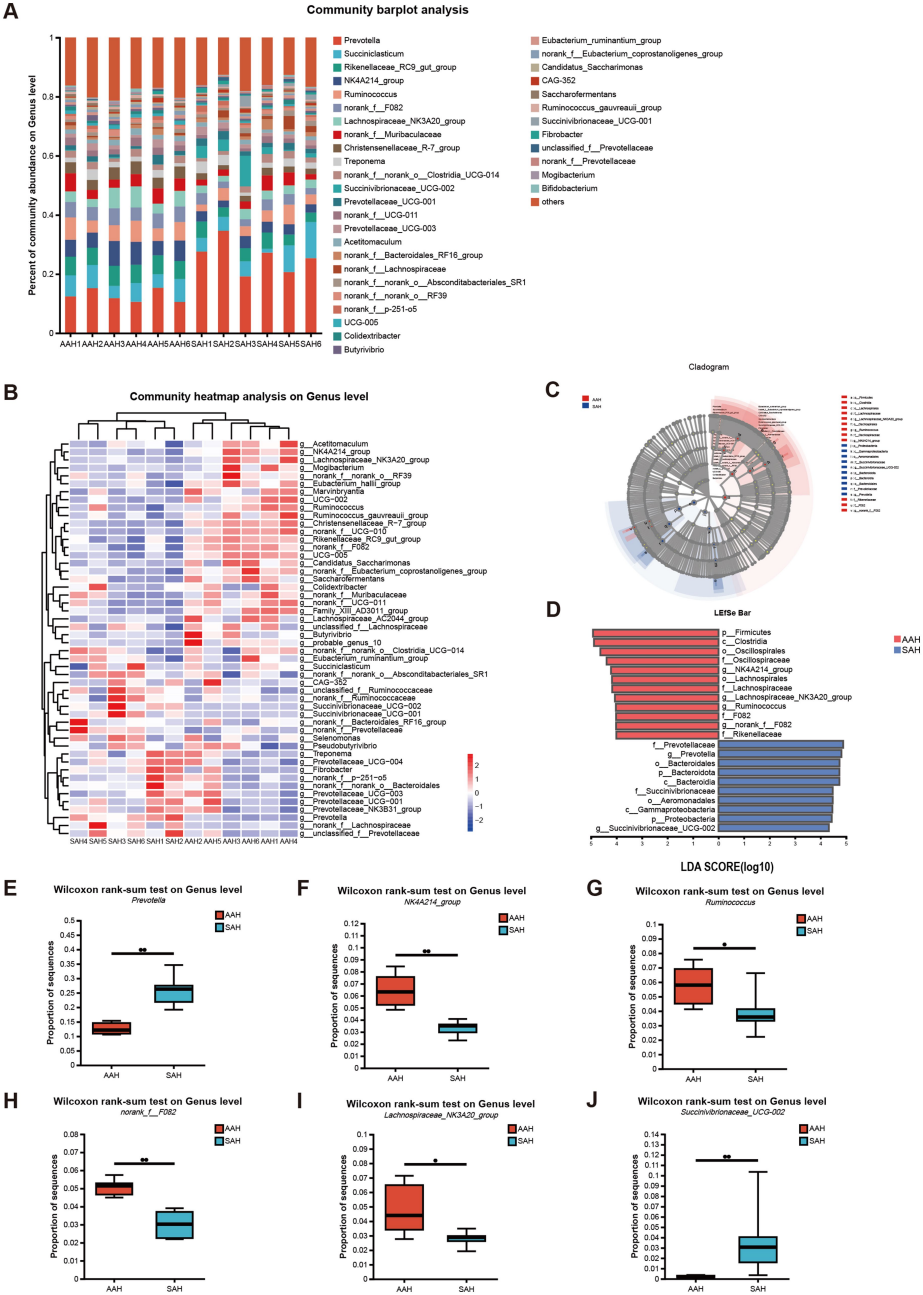
Analysis of the composition and difference of the rumen microbiota of dairy cows. AAH, American alfalfa hay; SAH, Spanish alfalfa hay. **(A)** Community bar plot analysis at the genus level. **(B)** Community heatmap analysis of 50 species at the genus level. **(C,D)** Linear discriminant analysis (LDA) effect size (LEfSe) analysis of differential enrichment of the ruminal microbes at the genus level: **(E)** *Prevotella*; **(F)** *NK4A214_group*; **(G)** *Ruminococcus*; **(H)** *norank_f_F082*; **(I)** *Lachnospiraceae_NK3A20_group*; **(J)** *Succinivibrionaceae_UCG-002*. AAH, American alfalfa hay; SAH, Spanish alfalfa hay. * 0.01 < *p* ≤ 0.05. ** 0.001 < *p* ≤ 0.01.

The five most abundant genera in the rumen of AAH group and SAH group were *Prevotella* (12.65 and 25.76%), *Succiniclasticum* (6.31 and 6.18%), *Rikenellaceae_RC9_gut_group* (6.27 and 4.26%), *NK4A214_group* (6.46 and 3.30%) and *Ruminococcus* (5.77 and 3.29%), respectively (Figure 4A). Microbiota profiles in the AAH group and the SAH group showed distinct clustering patterns. Notably, most dominant genera in the AAH group were from the *Bacillota phylum*, whereas those in the SAH group predominantly belonged to the *Bacteroidota phylum* (Figure 4B). Further analysis using LEfSe and linear discriminant analysis revealed several genera with significantly different abundances between the two groups (Figures 4C,D). Specifically, cows in the SAH group showed a significant increase in the relative abundance of *Prevotella* (Figure 4E) and *Succinivibrionaceae_UCG-002* (Figure 4J) (*p* < 0.05), while the abundance of *NK4A214_group* (Figure 4F), *Ruminococcus* (Figure 4G), *norank_f_F082* (Figure 4H), and *Lachnospiraceae_NK3A20_group* (Figure 4I) (*p* < 0.05) was significantly reduced compared to the AAH group.

### 3.5 Influence on the correlation between ruminal microbes and DMI, milk yield, and milk composition

**Figure 5:**
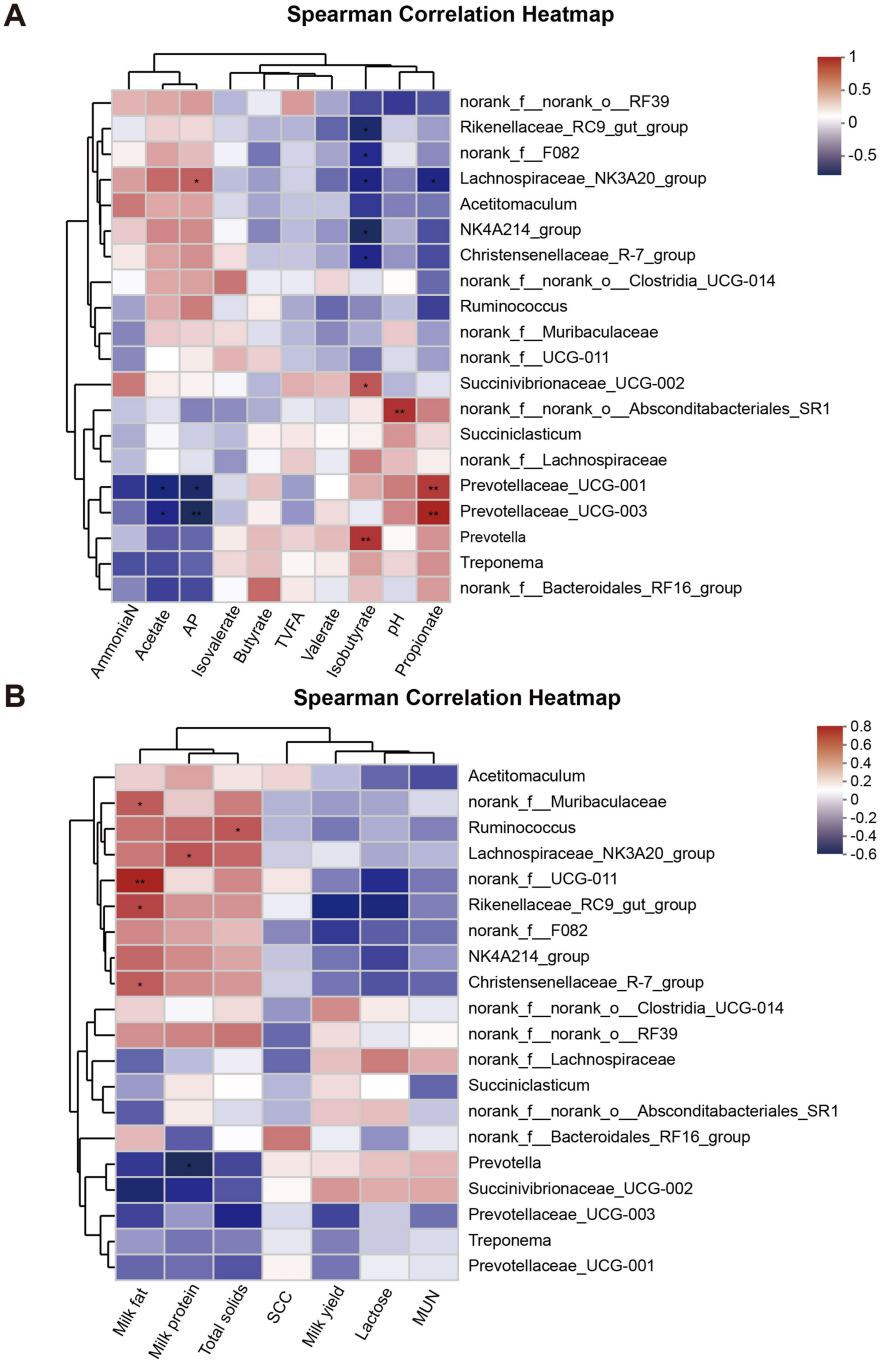
Analysis of the correlation between the rumen microbiota and milk yield, milk composition, and rumen fermentation parameters. **(A)** Correlation analysis of rumen microbiota with milk yield and milk composition. **(B)** Correlation analysis of rumen microbiota with rumen fermentation parameters. * 0.01 < *p* ≤ 0.05; ** 0.001 < *p* ≤ 0.01.

The Spearman correlation analysis revealed that *Prevotella* and *Succinivibrionaceae_UCG-002* were significantly positively correlated with isobutyrate, while *Christensenellaceae_R-7_group*, *Lachnospiraceae_NK3A20_group*, *norank_f_F082*, *NK4A214_group*, and *Rikenellaceae_RC9_gut_group* were negatively correlated with isobutyrate (*p* < 0.05). Additionally, *Lachnospiraceae_NK3A20_group* showed a significant positive correlation with A/P (*p* < 0.05), whereas *Prevotellaceae_UCG-003* and *Prevotellaceae_UCG-001* were positively correlated with propionate (*p* < 0.05) but negatively correlated with A/P (*p* < 0.05, Figure 5A). Moreover, the analysis indicated that milk fat was significantly positively associated with *norank_f_UCG-011*, *Christensenellaceae_R-7_group*, *norank_f_Muribaculaceae*, and *Rikenellaceae_RC9_gut_group* (*p* < 0.05). In contrast, *Prevotella* exhibited a negative correlation with milk protein (*p* < 0.05), while *Lachnospiraceae_NK3A20_group* showed a positive correlation with milk protein (*p* < 0.05). *Ruminococcus* was found to have a significant positive correlation with total solids (*p* < 0.05). However, no significant correlation was observed between MY and rumen microorganisms (*p* > 0.05, Figure 5B).

### 3.6 PICRUSt predictions of ruminal microbial functions

**Figure 6:**
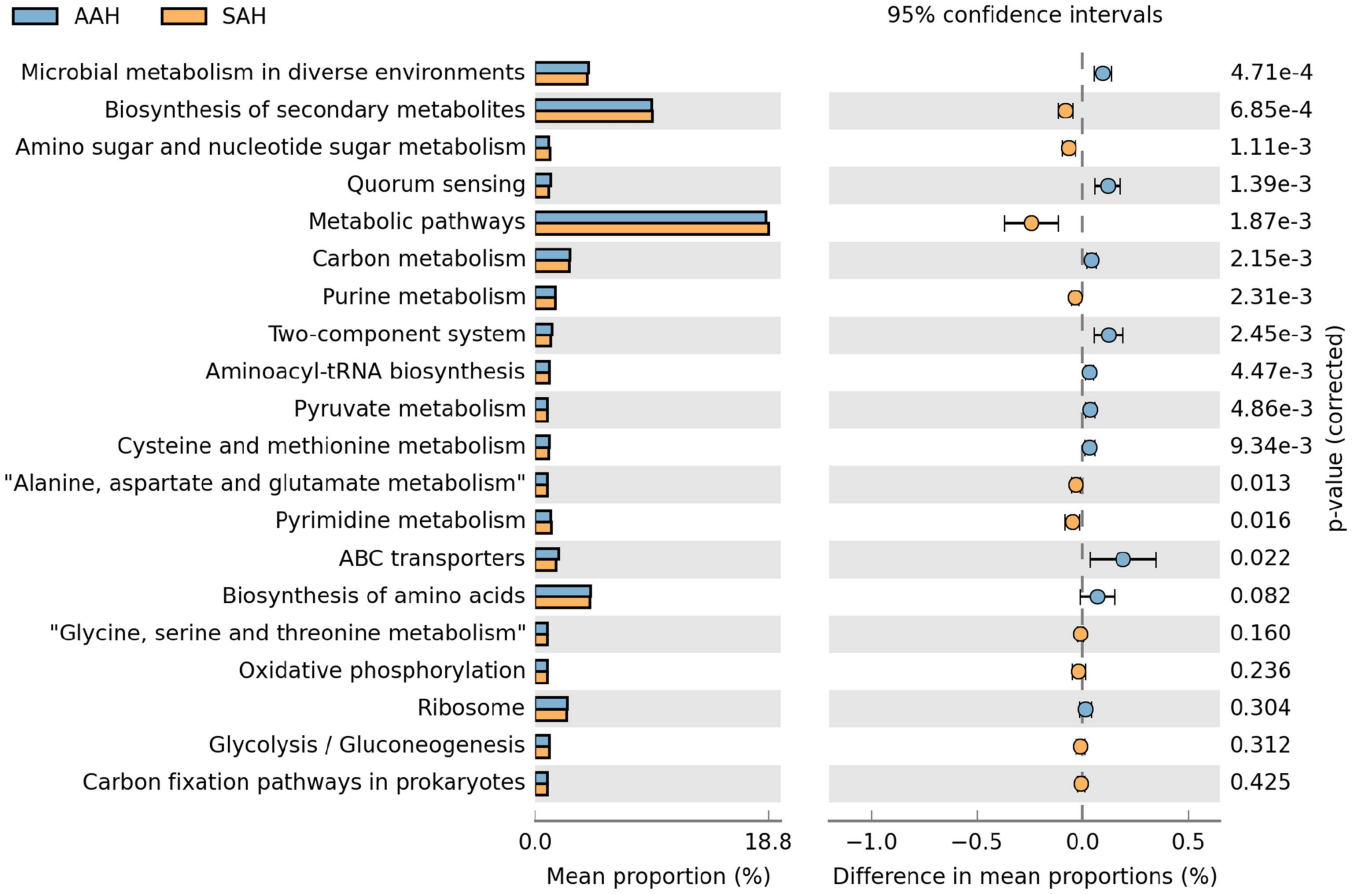
The top 20 metabolic pathways involved by rumen microbes in the two groups of dairy cows at KEGG level 3. AAH, American alfalfa hay; SAH, Spanish alfalfa hay.

Based on the KEGG database, functional predictions of rumen microorganisms were obtained using PICRUSt and analyzed with STAMP software. The top 20 metabolic pathways with the highest functional abundance were identified for comparison between the two groups (Figure 6). The analysis revealed that 7 metabolic pathways were significantly more abundant in the SAH group compared to the AAH group. These pathways included biosynthesis of secondary metabolites, amino sugar and nucleotide sugar metabolism, general metabolic pathways, purine metabolism, alanine, aspartate, and glutamate metabolism, and pyruvate metabolism. Conversely, eight metabolic pathways were notably reduced in the SAH group relative to the AAH group. These pathways included microbial metabolism in diverse environments, quorum sensing, carbon metabolism, two-component systems, aminoacyl-tRNA biosynthesis, pyrimidine metabolism, cysteine and methionine metabolism, and ABC transporters.

## 4 Discussion

Imported alfalfa hay from America is widely used in the Chinese dairy industry. Recently, SAH has become an important alternative source to stabilize the alfalfa hay supply market, particularly in the wake of the COVID-19 pandemic. Despite its utility, there are concerns over the potential impacts of SAH on MY and milk quality, which are crucial factors influencing dairy farmers’ income. Thus, more research is needed to assess its dietary effects. This study aimed to compare the feeding effects of two types of imported alfalfa hay: SAH and AAH, by providing both hays to mid-lactation cows at a 12.88% dietary dry matter inclusion for a period of 60 days. Each hay was designed to provide the same nutritional level to ensure a fair comparison.

After 60 days of continuous monitoring on 360 dairy cows, the MY of AAH-cows and SAH-cows changed from 21.52 kg/d and 22.48 kg/d to 21.00 kg/d and 21.98 kg/d, respectively, which was in line with the expected MY fluctuations in mid-lactation cows (23). Similarly, there were no significant fluctuations in DMI between the two groups, with AAH-cows consuming approximately 22.24 kg/day and SAH-cows consuming 22.47 kg/day. In addition, milk samples were collected from 12 representative cows in each group to assess milk composition. The analysis showed no significant differences in milk fat percentage, milk protein percentage, or overall milk composition between the SAH and AAH groups. These results indicate that the effects of SAH on MY and milk composition are comparable to those of AAH. This conclusion is supported by the lack of significant differences in nutrient digestibility between the two groups.

To further explore the feeding effects of AAH and SAH, the representative indicators reflecting energy, protein and lipid metabolism in the serum were detected in this study, which were not affected by the different sources of alfalfa hay. In addition, there were no significantly differences in the activity of alanine aminotransferase and aspartate aminotransferase, which are sensitive indicators of liver metabolism and heart health in animals. Previous research has reported serum concentrations of glucose and urea in mid-lactation cows to be around 3.2 and 4.6 mmol/L, respectively (23). The results in this study align with these findings, despite variations that can be influenced by the animal diet (24). These results suggest that different sources of alfalfa hay do not affect the normal physiological condition of dairy cows during mid-lactation, further supporting the similarity in feeding effects between AAH and SAH.

To further compare the effects of SAH and AAH on dairy cows, rumen fermentation parameters were determined in this experiment. The rumen, a specialized digestive organ in ruminants, plays a critical role in the digestion process due to its large population of microorganisms (25). In the rumen, carbohydrates are degraded to produce VFA, such as propionic acid, acetate, and butyric acid, which are the primary productive substances and make up 70–80% of the total energy (26). In this study, the ruminal pH values for both groups of cows ranged from 6.51 to 6.56, which are conducive to the growth of fibrolytic bacteria and effective fiber digestion (27). The results showed that there were no significant differences in pH or total VFA concentrations between the two groups. The stability of the rumen micro-ecology in adult ruminants may be the reason for this finding (28, 29). Additionally, the ratio of acetic acid to propionic acid, an important indicator of rumen fermentation efficiency (30), was found to be similar between the two groups of dairy cows in the current study, indicating that rumen fermentation was not affected by either alfalfa hay type. Furthermore, ruminal NH_3_-N, a marker of protein breakdown and microbial protein synthesis (31), showed no significant differences between the groups. This result aligns with the digestibility of crude protein. In summary, SAH did not cause changes in rumen fermentation parameters.

To demonstrate the abundance of microbial species in the rumen fluid samples, a rarefaction curve was constructed between the quantity of sequences obtained through random sampling and the Shannon indices of diversity. A flat curve indicated that the sequencing depth was sufficient and had captured most of the OTUs. Furthermore, we evaluated the alpha diversity to determine if different sources of alfalfa hay influenced microbial diversity in the rumen. The Chao1 index, which reflects the total number of species (32), showed significant differences between the two groups. The rumen microbial communities in the AAH and SAH groups were significantly different according to the β-diversity analysis. These findings indicate that the source of alfalfa hay affected the abundance, composition, and structure of the rumen microbial community.

In this study, 16S rRNA sequencing revealed that *Bacteroidota* and *Bacillota* had the greatest percentage of bacteria in the ruminal flora at the *phyla* taxonomic level (33, 34). *Bacteroidota* are primarily involved in breaking down intricate macromolecular organic materials, such as the conversion of carbohydrates into monosaccharides. Meanwhile, *Bacillota* produce extracellular enzymes, including proteases, lipases, and cellulases, which help hydrolyze proteins, lipids, amino acids, and hemicellulose (35). At the phylum level, *Bacillota* and *Bacteroidota* were the dominant bacterial groups in the rumen in this study. Specifically, the relative abundance of *Bacillota* was 56.35 and 40.88% in the AAH and SAH groups, respectively, while *Bacteroidota* constituted 35.99 and 46.64% in these groups. These findings are in line with findings from previous studies (16).

Ruminal microecological studies have proposed the existence of a core ruminal microbiome and have reported significant changes in the abundance of core bacterial genera between animals (36). Due to differences in diet, days in milk, parity, and sample size, the core rumen microbes can differ across studies to varying extents (37). *Prevotella* and *Succinivibri-onaceae_UCG-002*, which are more abundant in the SAH group at the genus level in the rumen, may be the core differential bacteria. *Prevotella*, belonging to the *Bacteroidota*, can decompose plant proteins, peptides, hemicellulose and pectin into acetic acid, succinic acid and a small amount of isobutyrate, which are directly utilized by dairy cows (16). According to Calabrò et al. (38), the higher *Provetella* content in the SAH group could be due to the higher hemicellulose content in this study. *Succinivibrionaceae_UCG-002*, belonging to the *Bacillota*, is a typical fiber-degrading bacterium that breaks down fiber and cellobiose into succinic acid, acetic acid and carbon dioxide (39). Correlation analysis revealed a significant positive relationship between the abundance of *Prevotella* and *Succinivibrionaceae_UCG-002* and the isobutyrate molar proportion in the rumen. The SAH group exhibited a higher isobutyrate molar proportion compared to the AAH group. In addition, *NK4A214_group*, *Ruminococcus*, *norank_f_F082* and *Lachnospiraceae_NK3A20_group* were more prevalent in the AAH group and might represent the core differential bacteria in this study. *Ruminococcus* and *NK4A214_group*, belonging to the *Bacillota*, are beneficial bacteria in the rumen, efficiently degrading starch and fiber, respectively, and producing VFAs to provide energy to the animal (40). *Norank_f_F082* and *Lachnospiraceae_NK3A20_group* are similarly the main components of rumen microbiota in ruminates and are closely related to VFA production (41, 42). In this study, *NK4A214_group*, *Ruminococcus*, *norank_f_F082* and *Lachnospiraceae_NK3A20_group* were significantly negatively correlated with isobutyrate molar proportion. However, these core differential microorganisms were not significantly correlated with total VFA, molar proportions of acetate and propionic acid, and MY. In fact, except for the isobutyrate molar proportion, there were no other significant differences between the two groups in the rumen fermentation parameters. This indicated that changes in these core microorganisms did not alter ruminal fermentation patterns, which is one of the reasons why different sources of alfalfa hay did not cause changes in cow performance. Future studies could explore combining alfalfa hay from various sources with other feed ingredients to enhance efficiency by altering rumen microbial composition and improving dairy cow feed efficiency, which is an approach to refined feeding.

We investigated the impact of different alfalfa hay sources on ruminal microbial metabolic pathways in dairy cows. Using STAMP software to analyze microbial functions, we found notable differences between the two groups. Feeding SAH significantly up-regulated functions related to the biosynthesis of secondary metabolites, amino sugar and nucleotide sugar metabolism, metabolic pathways, purine metabolism, alanine, aspartate, and glutamate metabolism, as well as pyruvate metabolism. Conversely, feeding AAH led to significant up-regulation of microbial metabolism in diverse environments, quorum sensing, carbon metabolism, two-component systems, aminoacyl-tRNA biosynthesis, pyrimidine metabolism, cysteine and methionine metabolism, and ABC transporters. Despite these differences in microbial composition and metabolic functions, there were no changes in MY, DMI, blood biochemical parameters, or ruminal fermentation patterns between the two groups. Further investigation is needed to understand and verify the reasons behind these findings.

## 5 Conclusion

We found that the SAH and AAH with the similar nutritional levels had the same feeding effects, as evidenced by no significant differences in MY, milk composition, blood biochemical parameters, or rumen fermentation parameters between the two groups of dairy cows. However, the relative abundances of *Prevotella*, *Succinivibrionaceae_UCG-002*, *NK4A214_group*, *Ruminococcus*, *norank_f_F082* and *Lachnospiraceae_NK3A20_group* were changed significantly between the two groups. Despite these changes, no significant correlation was found between these microbial variations and milk yield. These findings provided reference for the application of alfalfa hay from different sources, the improvement of economic benefit of dairy farm and expansion of the alfalfa supply market.

## Data Availability

The datasets presented in this study can be found in online repositories. The names of the repository/repositories and accession number(s) can be found in the article/supplementary material.

## Footnotes

^1^ https://cloud.majorbio.com/page/tools/

^2^ http://www.genome.jp/kegg/
